# Is It Attachment Style or Socio-Demography: Singlehood in a Representative Sample

**DOI:** 10.3389/fpsyg.2015.01738

**Published:** 2015-11-10

**Authors:** Katja Petrowski, Susan Schurig, Gabriele Schmutzer, Elmar Brähler, Yve Stöbel-Richter

**Affiliations:** ^1^Department of Psychotherapy and Psychosomatic Medicine, University Hospital Carl Gustav Carus, Technische Universität Dresden, Dresden, Germany; ^2^Department of Medical Psychology and Medical Sociology, University of Leipzig, Leipzig, Germany; ^3^Department Psychosomatic Medicine, University of Mainz, Mainz, Germany

**Keywords:** adult attachment scale, attachment style, partnership status, representative sample, single person

## Abstract

Since the percentage of single adults is steadily increasing, the reasons for this development have become a matter of growing interest. Hereby, an individual’s attachment style may have a connection to the partnership status. In the following analysis, attachment style, gender, age, education, and income were compared in regard to the partnership status. Furthermore, an analysis of variance was computed to compare the attachment style within different groups. In 2012, a sample of 1,676 representative participants was used. The participants were aged 18 to 60 (*M* = 41.0, SD = 12.3); 54% of the sample were female, and 40% were single. Attachment-related attitudes were assessed with the German version of the adult attachment scale (AAS). Single adult males did not show a more anxious attachment style than single adult females or females in relationships. Younger, i.e., 18 to 30 years old, paired individuals showed greater attachment anxiety than single individuals, whereby single individuals between the ages of 31 to 45 showed greater attachment anxiety than individuals in relationships. In addition, single individuals more frequently had obtained their high school diploma in contrast to individuals in relationships. Concerning attachment style, the individuals who had not completed their high school diploma showed less faith in others independent of singlehood or being in a relationship. Concerning age, older single individuals, i.e., 46 to 60 years, felt less comfortable in respect to closeness and showed less faith in others compared to paired individuals. Logistic regression showed that individuals were not single if they did not mind depending on others, showed high attachment anxiety, were older, and had lower education. An income below € 2000/month was linked to a nearly 13-fold increase of likelihood of being single. In sum, the attachment style had a differential age-dependent association to singlehood versus being in a relationship. Education played also a role, exclusively concerning faith in others.

## Introduction

Due to the wide variety of living styles and increasing individualization, there is an ever growing number of single adults in highly industrialized countries such as Germany. In 1999, the percentage of single adults in Germany (who do not live in a partnership) was 17.8%, which increased to 20.2% in 2011 (Federal Statistical Office Germany, 2013a). Similar figures were reported by the U.S. Census Bureau. The proportion of single individuals increased by 10 percentage points from 17 to 27% between 1970 and 2012 (Vespa et al., 2013). These numbers may not replicate the actual life situation precisely as only the legal family status was assessed. However, singlehood is an important way of life to investigate as it is connected to lower physical health (Müller et al., 1999; Lammintausta et al., 2013) and less psychological well-being (Mookherjee, 1997; Brown, 2000; Scharfe and Cole, 2006).

### Social Changes and Relationship Status

The increasing numbers of single adults may be explicable by a change in values toward individualism, independency, and opting for a career (Federal Statistical Office Germany, 2012a). Career orientation and fulfillment in a career require greater mobility and flexibility at work due to globalization, which in turn tends to lead toward a singlehood society (Schulz, 2008; Federal Statistical Office Germany, 2012a). These developments do not only apply to men but to women as well since, nowadays, women are more highly educated in general, thus holding higher positions than ever before (Nave-Herz, 2001; Wagner-Link, 2005). Besides, the change in life style and an increase in singlehood can also be explained by individual life expectancy. In addition, prosperity is rising while birthrates are dropping (Pötzsch, 2012).

These changes in society are reflected in the socio-demographic characteristics of singlehood. Modern single adults are rather young (18–34 years; Federal Statistical Office Germany, 2012b), tend toward an independent life-style over having children (Federal Statistical Office Germany, 2012b), and have a degree in higher education, especially the women (Knußmann, 2007). Seventy-four percent of single male adults and 72% of the single female adults work fulltime (Federal Statistical Office Germany, 2012a), and most of the single adults (56%) live in large urban areas (Federal Statistical Office Germany, 2012a). Unfortunately, the changes in values toward individualism and a career cannot serve as a complete explanation based on statistical numbers. The attachment theory, however, may provide one possible answer. Therefore, research focused on the attachment theory and singlehood already several years ago (DePaulo, 2006; Schachner et al., 2008).

### Attachment Theory and Relationship Status

The attachment theory (Bowlby, 1973) postulates that through early experiences with their primary caregiver individuals develop an internal working model of themselves, others, and close relationships. Based on the internal working model, individuals develop beliefs about themselves (worthy of care, love, and attention) as well as about significant others (dependable, responsible, and trustworthy). These beliefs influence expectations, perceptions, and behavior in future relationships. They are also responsible for the choice of a partner as well as the secure, avoidant, and anxious/ambivalent attachment style in close relationships (Hazan and Shaver, 1987; Collins and Read, 1994).

Individuals with a secure attachment style show positive beliefs about themselves (e.g., self-worth, social competence, sense of control) and about their partner or others (e.g., trustworthy, dependable, and altruistic). On the other hand, individuals with an anxious/ambivalent attachment style can be characterized by negative beliefs about themselves but positive views of the partner or others as well as an obsessive preoccupation with their partner. Individuals with an avoidant attachment style have a positive view of themselves and a negative view of their partner and others. They show a fear of intimacy and a lack of acceptance of the partner as well as distrust of others.

Since the attachment style influences the perception of others and the selection of future partners, it might also influence the partnership status (Hazan and Shaver, 1987). (1) One attachment-specific explanation of singlehood may be that these individuals have a more avoidant attachment style than paired individuals. Avoidant-attached individuals favor independence as well as self-reliance and have a need for a sense of distance and autonomy (DePaulo, 2006). (2) Another perception of singlehood might be that these individuals show a more anxious attachment style than paired individuals. Anxious-attached individuals have been rejected by relationship partners who would not accept their anxiety, clinginess, and intrusiveness. In addition, (3) one might also argue that single adults may have an attachment figure other than a romantic partner or a parent to rely upon (DePaulo, 2006). Doherty and Feeney (2004) showed that single individuals usually named their mother or a friend as primary attachment figure. Therefore, single adults might be as securely attached as paired individuals.

### Previous Research

However, these deflections are exclusively derived from a theoretical point of view. To our knowledge, until now there have been only a few studies that investigated the association of attachment style and relationship status that contradict each other. Comparing the relationship status in a longitudinal study, on the one hand, an association between attachment style and the relationship status 4 years later could be shown (Kirkpatrick and Hazan, 1994). *Securely* attached adults were most likely married. Whereas *avoidant* individuals were most likely single and not looking for a partner or only for a casual relationship (see also longitudinal Kirkpatrick and Hazan, 1994; Schindler et al., 2010); *ambivalent/anxious* attached adults were also most likely single but seeking a partner. These results were supported by Schindler et al. (2010). However, the differences in the different single subgroups cannot be replicated when cross-sectionally serious, casual, and non-dating young adults were compared (Bookwala, 2003). Higher scores on fearful-anxious attachment significantly increased the odds for being single (Bookwala, 2003; Adamczyk and Bookwala, 2013). Due to the insecure attachment style singles reported feeling less comfortable with closeness and intimacy, more problems with depending on others, and more worries about being unloved or fear of rejection (Adamczyk and Bookwala, 2013).

On the other hand, Schachner et al. (2008) found no significant differences between single and paired individuals in the prevalence of insecure attachment. This study, however, showed a socio-demographic attachment style specificity. In contrast to the attachment theory attachment *anxiety* was associated with singlehood in *men*. Concerning gender effects on attachment, studies showed diverse results. Parent–infant attachment studies using the Adult Attachment Interview did not find any differences between males and females (see van IJzendoorn, 2000; Bakermans-Kranenburg and van IJzendoorn, 2009a, 2010). However, one study reported that girls were more securely attached compared to boys who were more avoidant attached (Bakermans-Kranenburg and van IJzendoorn, 2009b). For romantic attachment relationships a meta-analysis showed higher anxiety and lower avoidance in females than in males (Del Giudice, 2011). In sum, gender differences in close relationships may be present, but since an anxious attachment style in single males was observed only in a small sample, these results need to be replicated in a larger and more representative sample.

### Research Questions

Even though singles show a higher probability for insecure attachment compared to coupled individuals, inconsistencies have been published for the second classification (anxious versus avoidant). Therefore, a larger representative sample is necessary in order to clarify the second classification.

Even though the socio-demographic characteristics for single adults have already been reported, education and age have not yet been investigated in the context of singlehood and attachment style. Differences in the attachment style between singles and individuals in partnership might be due to differences in socio-demographics.

### Present Study

Since attachment style and socio-demographic variables are associated (Schachner et al., 2008; Del Giudice, 2011) and a representative study on the relationship status and attachment style is missing, the aim of the present study was to analyze attachment style and socio-demographic variables in a large representative sample.

First, the findings of Schachner et al. (2008) were replicated. Therefore one hypothesized that single males show a more anxious attachment style than single females or females in relationships (H1). Second, the avoidant attachment style changed at an older age depending on the relationship status (Petrowski et al., 2012). Therefore, one hypothesized that older single adults show a more avoidant attachment style compared to individuals in a relationship at an older age, (H2). Third, since single adults show higher education and intelligence as well as scholastic achievement, which is associated with attachment security (Bowlby, 1973; Wacha, 2010), more attachment security might be observable in more highly educated single adults as well (H3). In addition, variables that might significantly predict singlehood were analyzed.

## Materials and Methods

### Description of Participants

In 2012, the USUMA (Unabhängiger Service für Umfragen, Methoden und Analysen) of the Berlin Polling Institute selected households and participants by random-route sampling (Arbeitsgemeinschaft ADM-Stichproben and Bureau Wendt, 1994). Sixty-two percent of all contacted individuals filled out the questionnaire. Of these, the final sample of *N* = 2,510 persons was examined. Using information from the Federal Statistical Office, the final sample was approved to be truly representative for the German residential population in 2012. All the participants volunteered and received a data protection declaration in agreement with the Helsinki Declaration. The participants ranged in age from 14 to 91 years (*M* = 49.4, SD = 18.0); 53.4% of the sample were female. Forty-two percent of the sample were single, and 58% were in a relationship. Concerning education, 83% had no high school diploma and 17% did. Forty-seven percent of the participants earned below € 2000 per month, 50% earned € 2000 or more. An additional 3% gave no response.

In order to minimize the effect of widowhood (see Federal Statistical Office Germany, 2013b) for the analysis, a subsample of 1,676 participants was used, aged 18 to 60 (*M* = 41.0, SD = 12.3). Fifty-four percent of the subsample were female; 40% were single, and 60% were in a relationship. Concerning education, 80% had no high school diploma and 20% did. Forty percent of the participants earned below € 2000 per month, and 60% earned € 2000 per month, or more.

The study was approved in accordance with the ethics guidelines of the “German Professional Institution for Social Research” (Arbeitskreis Deutscher Markt- und Sozialfor-schungsinstitute, Arbeitsgemeinschaft Sozialwissenschaftlicher Institute; Berufsverband Deutscher Markt- und Sozialforscher).

### Instruments

Attachment-related attitudes were assessed with the German version of the original adult attachment scale (AAS; Collins and Read, 1994). The participants scored the 18 items, using a five-item Likert-type scale with values ranging from “*not at all*” to “*very*.” Factor analysis led to three main factors (each consisting of six items): capacity to feel close to the partner (close; “I find it relatively easy to get close to people.”); capacity to depend on others (depend; “I find it difficult to allow myself to depend on others.”); and anxiety of losing an intimate partner (anxiety; “I often worry that romantic partners don’t really love me.”). The reliability of all three scales was satisfactory (α = 0.72–0.79; Schmidt et al., 2004). A high value on the scale “close” means a person is comfortable with closeness and intimacy. A high value on the scale “depend” symbolizes no problems with dependency, and a high value on the scale “anxiety” means a person often worries about being unloved. The adult attachment could be measured based on scores on the three subscales. The secure attachment is characterized by high scores on AAS subscales “close” and “depend” and a low score on AAS subscale “anxiety.” The avoidant attachment is characterized by low scores on all three subscales. The anxious attachment is characterized by a high score on the AAS subscale “anxiety” and moderate scores on the subscales “close” and “depend.”

The information on being in a relationship (Item: “Do you live in a relationship”) was based on self-report data and need not represent the legal family status. Furthermore, it was insured that the single individuals were not married and did not live in a partnership.

### Statistical Procedure

For the analysis the Statistical Package for the Social Science (SPSS) in version 20.0 was used.

Since the sample size was large, the significance level was corrected for the sample size. For the descriptive analysis of the individuals with a different relationship status and to answer the first hypothesis, χ^2^-tests for independent samples and one-way analyses of variance were used. To test the requirements, the Levene test for variance homogeneity and the Kolmogorov-Smirnov test for normal distribution were implemented. There was no evidence that the requirements were not met. Since the large sample size leads to significant results more easily, effect sizes were calculated. A Cohen’s *d* ≥ 0.20 is a small but relevant effect, Cohen’s *d* ≥ 0.50 is a moderate effect, and Cohen’s *d* ≥ 0.80 is a strong effect (Cohen, 1988). A Cramer’s φ = 0.1 is a small but relevant effect, Cramer’s φ = 0.3 is a moderate effect, and Cramer’s φ = 0.5 is a strong effect (Sedlmeier and Renkewitz, 2008).

In order to answer the hypothesis about the influence of education, income, and relationship status on the adult attachment style (H2 and H3), a three-way analysis of variance was implemented. As effect size, the Partial Eta-Square η^2^ was calculated. A η^2^ = 0.01 is a small effect, η^2^ = 0.06 is a mild effect, and η^2^ = 0.14 is a strong effect.

In order to be able to predict singlehood, a binary logistic regression analysis was used (coupled vs. single, coded as *coupled* = 0 and *single* = 1). The gender of the interviewee, age, education and income as well as the scales of Adult Attachment “close,” “depend,” and “anxiety” were included stepwise in the logistic regression analysis. Of course, even though it is not certain if attachment style developed before the individual relationship status, these variables were treated as predictors.

## Results

In the following analysis, attachment style, gender, age, education, and income were compared concerning partnership status.

### Socio-Demography and Relationship Status

Concerning *gender*, 46% of the singles were male and 54% were female whereas 46% of the individuals in a partnership were male and 54% female. According to the hypothesis H1 a gender effect was expected but this gender difference was not significant [χ^2^ (1, *N* = 1676) = 0.06, *p* > 0.05; Cramer’s φ = 0.01]. The single individuals had a mean *age* of *M* = 37.2 (SD = 13.3) compared to the individuals in a partnership with a mean age of *M* = 43.5 (SD = 10.9). Individuals in a partnership were mostly between 31 and 60 years old whereas the single individuals were 18 to 30 respectively 46 to 60 years old. This difference was highly significant [χ^2^ (2, *N* = 1676) = 133.94, *p* > 0.001] with a Cramer’s φ = 0.28, which meant a small (almost moderate) effect. According to the hypothesis H3 it was expected that the singles have a higher education. Twenty-five percent of the single individuals had a *high school diploma* compared to the individuals in a partnership with 17%. This difference was highly significant [χ^2^ (1, *N* = 1676) = 15.43, *p* < 0.001] with Cramer’s φ = 0.10, which meant a small but relevant effect. Concerning *income*, 71% of the single individuals earned below € 2000/month, and 29% earned € 2000/month, or more. In contrast, only 20% of the individuals in a partnership earned below € 2000/month whereas 80% earned € 2000/month, or more. This difference was highly significant [χ^2^ (1, *N* = 1632) = 429.74, *p* < 0.001] as well with Cramer’s φ = 0.51, which meant a strong effect.

Concerning *partnership status*, the individuals in a partnership showed significantly higher values on the AAS “depend” compared to the single individuals (see Table 1). Cohen’s *d* was 0.18, which meant a too small and not relevant effect. Regarding *gender* again an effect was expected (H1), but there were no significant differences on the AAS scales (Table 1). Concerning *age* the expectation was that an older age was connected to a more avoidant attachment style (H2). The age group of 46 to 60 significantly showed the lowest values on the AAS “depend” compared to the other two age groups with η^2^ = 0.01, which meant a small but relevant effect (see Table 2).

**TABLE 1:** **Differences for Adult Attachment Scale by comparing marital status, gender, education, and income (***N*** = 1676)**.

	**Partnership**		**No Partnership**				
**Variable**	***M***	**SD**	***M***	**SD**	**df**	***t***	***d***
AAS Close	3.71	0.66	3.70	0.66	1674	0.40	0,02
AAS Depend	3.68	0.76	3.53	0.81	1673	3.86***	0,18
AAS Anxiety	2.01	0.78	2.06	0.79	1643	-1,35	0,07
	**Male**		**Female**				
AAS Close	3.68	0.66	3.72	0.66	1674	-1.18	0,06
AAS Depend	3.61	0.77	3.63	0.79	1673	-0.58	0,03
AAS Anxiety	1.99	0.77	2.05	0.79	1643	-1.55	0,08
	**High school diploma**		**No High school diploma**				
AAS Close	3.73	0.65	3.70	0.66	1674	-0.79	0,05
AAS Depend	3.75	0.76	3.58	0.78	1673	-3.54***	0,17
AAS Anxiety	2.00	0.82	2.03	0.77	1643	0.57	0,04
	**Income below 2000 €**		**Income 2000 € and more**				
AAS Close	3.70	0.67	3.71	0.66	1630	-0.10	0,01
AAS Depend	3.50	0.79	3.70	0.76	1630	-5.08***	0,26
AAS Anxiety	2.11	0.79	1.97	0.77	1600	3.54***	0,18

***p <.001. d ≥ 0.20 small effect.

**TABLE 2:** **One-way analyses of variance (ANOVA) for effects of age (18–30; 31–45; 46–60) on the Adult Attachment Scale**.

	**Age 18–30**		**Age 31–45**		**Age 46–60**			***ANOVA***		**^**2**^**	
**Source**	***M***	**SD**	***M***	**SD**	***M***	**SD**	**df**	**SS**	**MS**	**F**	
AAS Close	3.74	0.65	3.72	0.66	3.67	0.66					
Between groups							2	1.49	0.74	1.71	0.00
Within groups							1673	727.34	0.43		
AAS Depend	3.70	0.78	3.65	0.76	3.54	0.79					
Between groups							2	8.12	4.06	6.71**	0.01
Within groups							1672	1011.96	0.61		
AAS Anxiety	2.11	0.84	1.99	0.74	2.01	0.77					
Between groups							2	3.64	1.8	2.98	0.00
Within groups							1642	1002.05	0.61		

**p <.01. η^2^ = 0.01 small effect.

Regarding *education* it was expected that higher education was connected to a more secure attachment style (H3). The individuals with a high school diploma showed significantly higher values on the AAS “depend” compared to the individuals without a high school diploma (see Table 1). Cohen’s *d* was 0.17, which meant a too small and not relevant effect. The individuals with an *income* below € 2000/month showed significantly lower values on the AAS “depend” compared to individuals with an income higher than € 2000/month. This effect (Cohen’s *d* = 0.26) was small but relevant. These individuals showed also significantly higher values on the AAS “anxiety” compared to the individuals with the higher income (see Table 1). But this effect (Cohen’s *d* = 0.18) was too small and not relevant.

Due to the missing differences between the single individuals and the individuals in a partnership concerning gender, gender was no longer included in the other analyses. According to hypotheses 2 and 3 it was expected that older single adults show a more avoidant attachment style and that single adults with a higher education show a more secure attachment style.

**FIGURE 1:**
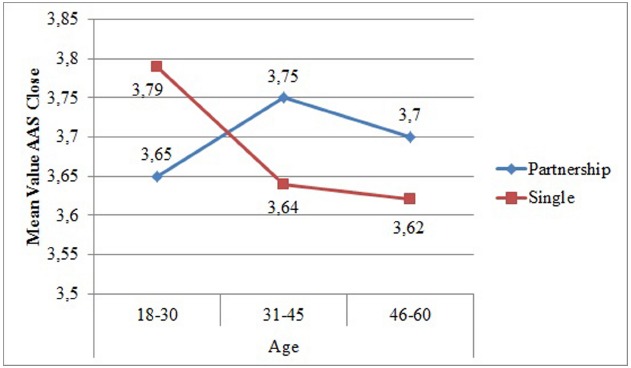
**Mean Values of Adult Attachment Scale Close depending on age and partnership**.

In the three-way-analysis of variance with partnership, age, and education as factors and the *AAS close* as continuous dependent variable, a small significant interaction effect between partnership and age could be shown [*N* = 1676, *F*(2, 1664) = 4.19, *p* = 0.015, η^2^ = 0.01]. Single individuals between the ages of 18 and 30 were more comfortable with closeness than coupled individuals similar in age. In contrast, single individuals of an older age felt less comfortable with closeness than the coupled individuals (see Table 3 and Figure 1). Education did not show an effect.

**TABLE 3:** **Means of Adult Attachment Scale for two-way interaction Partnership × Age**.

			**AAS Close**				**AAS Depend**				**AAS Anxiety**		
		**Yes**		**No**		**Yes**		**No**		**Yes**		**No**	
**Partnership**		***M***	**SD**	***M***	**SD**	***M***	**SD**	***M***	**SD**	***M***	**SD**	***M***	**SD**
Age	**18–30**	3.65	0.62	3.79	0.67	3.67	0.76	3.72	0.78	2.22	0.87	2.04	0.82
	**31–45**	3.75	0.66	3.64	0.66	3.74	0.74	3.46	0.79	1.94	0.72	2.12	0.77
	**46–60**	3.70	0.67	3.62	0.64	3.63	0.77	3.35	0.80	1.99	0.78	2.05	0.76

**FIGURE 2:**
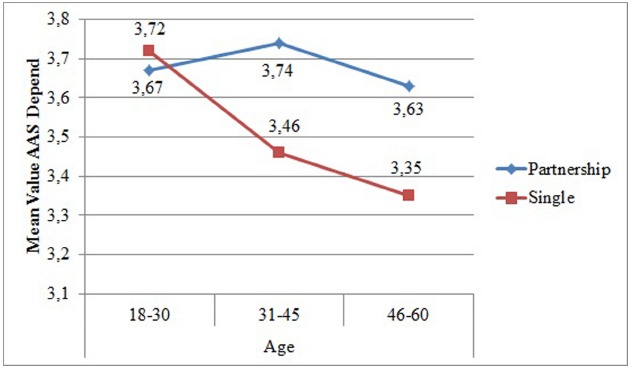
**Mean Values of Adult Attachment Scale Depend depending on age and partnership**.

In the three-way-analysis of variance with partnership, age, and education as factors and the *AAS depend* as continuous dependent variable (*N* = 1675), three small but relevant significant main effects of partnership, *F*(1, 1663) = 24.41, *p* < 0.001, η^2^ = 0.01, age, *F*(2, 1663) = 8.56, *p* < 0.001, η^2^ = 0.01, and education, *F*(1, 1663) = 11.92, *p* < 0.001, η^2^ = 0.01 were observed. Older individuals, single ones as well as individuals without a high school diploma showed less faith in others. Furthermore, the interaction between partnership and age, *F*(2, 1663) = 5.70, *p* = 0.003, η^2^ = 0.01, revealed that single individuals aged between 31 and 60 had less faith in others than coupled individuals (see Figure 2).

**FIGURE 3:**
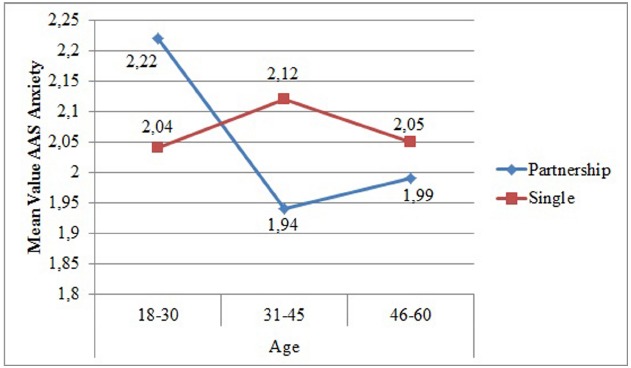
**Mean Values of Adult Attachment Scale Anxiety depending on age and partnership**.

In the three-way-analysis of variance with partnership, age, and education as factors and the *AAS anxiety* as continuous dependent variable, a significant interaction effect between partnership and age could be revealed (*N* = 1645), *F*(2, 1633) = 6.32, *p* = 0.002, η^2^ = 0.01. Coupled individuals at an age between 18 and 30 showed higher relationship anxiety than singles of the same age. In contrast, single individuals aged 31 to 45 are more anxious concerning relationships. This effect was small but relevant as well. Coupled and single individuals aged between 45 and 60 did not show any difference in relationship anxiety (see Figure 3).

### Predicting Singlehood

In order to be able to predict singlehood, age, education and income as well as the AAS scales “close,” “depend,” and “anxiety” were included stepwise (see Table 4). Since gender did not differentiate between the relationship statuses and adult attachment style in the χ^2^-test it was, therefore, not included.

**TABLE 4:** **Summary of stepwise Logistic Regression Analysis predicting Singlehood (n = 1602)**.

					**95% CI**		
**Variable**	***B***	**SE**	**Odds ratio**	**Wald statistic**	**Lower limit**	**Upper limit**	***Nagelkerkes R^2^***
***Step 1–3***							
Age	-0.05	0.01	0.95	81.55***	0.94	0.96	
Education^a^	-0.82	0.16	0.44	25.96***	0.32	0.60	.404
Income^b^	2.57	0.13	13.02	368.72***	10.02	16.92	
***Step 4***							
Age	-0.05	0.01	0.95	89.84***	0.94	0.96	
Education^a^	-0.88	0.16	0.42	29.08***	0.30	0.57	
Income^b^	2.56	0.14	12.88	354.31***	9.87	16.80	.414
AAS Close	0.02	0.13	1.02	0.02	0.79	1.31	
AAS Depend	-0.44	0.12	0.65	13.07***	0.51	0.82	
AAS Anxiety	-0.32	0.11	0.73	9.03**	0.59	0.90	

^a^no high school degree. ^b^under € 2000. **p <.01. ***p <.001.

The results of the logistic regression analysis showed that advanced age, not having a high school diploma, and high values on the scale “depend” as well as high values on the scale “anxiety” were connected with a decreased likelihood of being single. Whereas having an income below € 2000/month was linked to a nearly 13-fold increase of likelihood of being single. The total explained variance of being a single adult versus being in a partnership was 41.1%. The highest explained variance showed the income compared to the other socio-demographic variables (age and education) in the third step of the analysis. After adding the attachment scales to the analysis the explained variance increased only from 40.4 to 41.1%.

## Discussion

As the percentage of single adults in industrialized countries such as Germany and the USA is steadily increasing (Federal Statistical Office Germany, 2013a; Vespa et al., 2013), the reasons for this development are of growing interest. Besides the socio-demography, the attachment style might additionally explain the partnership status. In the following analysis, attachment style, gender, age, education, and income are compared in regard to the partnership status.

### Present Findings

Based on the present representative data, the single males did not show a more anxious attachment style than the single females or the females in relationships (H1). This contradicts the findings by Schachner et al. (2008) who postulated an association between attachment anxiety and singlehood in men. However, the younger coupled individuals, i.e., 18 to 30 years old, showed higher attachment anxiety than the single individuals. Furthermore, the single individuals aged between 31 and 45 showed higher attachment anxiety than the individuals in relationships. Thus, it is the age and not the gender that modulates attachment anxiety. This falsified the hypothesis 1.

The older singles, i.e., 46 to 60 years, showed a more avoidant attachment style (H2), felt less comfortable with closeness, and had less faith in others compared to the coupled individuals. Therefore, the second hypothesis can be accepted. In addition, the single individuals more often had a high school diploma in contrast to the individuals in relationships (H3). And concerning the attachment style, the individuals with a high school diploma had more faith in others and no problems with dependency independent of singlehood or being in a relationship. Therefore, hypothesis 3 can be accepted, as well.

### Interpretation

The singles in the representative study were generally younger than the coupled individuals which contradicts the findings of Schachner et al. (2008). Schachner et al. (2008) argued that the singles were older than the coupled individuals since the singles needed more time to find a partner but in case of the present study this is not possible. The younger singles in the present study might be explicable by the general changes in society.

Concerning attachment the *younger* single individuals feel more comfortable with closeness; have more faith in others and less attachment anxiety.

The present comfort with closeness and low rejection fear might be explicable by the attachment style present in sample of healthy individuals. These individuals show by the majority a secure attachment style (Fonagy et al., 1996). However, the *middle-aged* and *older* single individuals feel less comfortable with *closeness* compared to coupled individuals of the same age. This goes along with having less *faith in or depending less on* other individuals as well as higher attachment anxiety compared to coupled individuals. Therefore, single individuals do not show more avoidant or anxious attachment (H1/H2), or a specific attachment classification in general.

The older single individuals are less comfortable with closeness (more avoidant), show higher attachment anxiety, and have less faith in others. These results (Adamczyk and Bookwala, 2013) are in line with single individuals reporting lower levels of comfort with closeness, faith in others as well as higher levels of attachment anxiety compared with coupled participants.

The differences between the younger single individuals and the middle-aged single individuals might be explicable by attachment-specific events that may have occurred during the interim period. After the age of 30, these individuals left home, finished their education (Federal Statistical Office Germany, 2010) and built a partnership or a family as the developmental task (see the Developmental Task Theory, Havighurst, 1948). This developmental task might be connected to fear of failure (Arnett, 2000; Schulenberg et al., 2004) which would then lead to higher attachment anxiety.

During the interim periods, males have in average six to eight female sexual partners, and females have in average four male sexual partners (Mosher et al., 2005). Therefore, previously experienced partners or partnerships may be an influencing factor and may lead to lower levels of being comfortable with closeness, faith in others as well as higher levels of attachment anxiety.

The highly anxious individuals appeared also more distressed, and conflicts escalated more severe (Campbell et al., 2005). The anxiously-ambivalent attached individuals are unable to distance themselves from disappointing and conflictual relationships just as they are incapable of detaching themselves from overwhelming inner stress (Mikulincer and Orbach, 1995; see also Bowlby, 1973). Mikulincer and Orbach (1995) assumed that they seem to lack the control mechanisms needed to regulate inner distress as well as social relationships.

Piatkowski (2012) hypothesized that a woman needs a man for protection but that remaining single may also be a way of self-protection. In this situation, the single status followed upon an unsuccessful relationship, which made the individuals hesitant to start a new relationship (Piatkowski, 2012).

Individuals who worry about future relationships may attribute their worries to having been hurt in the past and not wanting to put themselves into a vulnerable position again, and, overall, they may believe that remaining single would protect them. Situations of this kind experienced by single individuals were not evaluated in the present study but should be considered in future research.

Anxious-attached middle-aged singles live through a longer single period after a previous relationship or to a longer period of singlehood without any previous relationships.

In addition, it cannot be distinguished whether the individuals even wish to have a new partner or choose singlehood as their life-style. The need for social contacts may be compensated by others than a romantic partner. However, these questions cannot be answered based on the present cross-sectional data as this would require longitudinal data. In future research, variables such as the desire for a romantic partner as well as the duration of singlehood should be taken into account, because whether single individuals live voluntarily or involuntarily without a partner is decisively connected to attachment anxiety or avoidance (Schmohr et al., 2010). So, voluntarily single individuals showed less attachment anxiety but higher attachment avoidance compared to involuntarily single individuals. In respect to their attachment avoidance, the involuntarily single individuals did not differ from individuals living in a partnership.

Besides age, education is associated with attachment in singlehood as well. The single individuals were more highly educated, and the more highly educated individuals showed fewer problems with dependency, which contradicts an anxious attachment style. Especially younger individuals showed strong career orientation, seeking fulfillment in a career (Federal Statistical Office Germany, 2012a). In addition, due to globalization, greater mobility and flexibility at work were required during recent years (Schulz, 2008; Federal Statistical Office Germany, 2012a). These developments did not only apply to men but to women as well since, nowadays, women are more highly educated in general, thus holding higher positions than ever before (Nave-Herz, 2001; Wagner-Link, 2005).

Concerning the individuals living in a partnership, younger coupled individuals showed higher attachment anxiety than single individuals of the same age. These results contrast the hypothesis that single adults experience higher attachment anxiety than couples (Schachner et al., 2008). Since the present data do not include information about relationship duration, it can only be presumed that the early days, weeks, or years of a relationship lead to insecurity and therefore attachment anxiety (Asendorpf, 2006; Scharfe and Cole, 2006). Possibly, after more assurance from being in the relationship, attachment anxiety decreases with the years (Asendorpf, 2006; Scharfe and Cole, 2006). In the present results, the coupled individuals of 31 years of age and older did not show high attachment anxiety. Since age effects on the attachment style are present in this respect, various studies have discussed the stability of the attachment style through the years (Schneider et al., 1998; Asendorpf and Wilpers, 2000; Zhang and Labouvie-Vief, 2004; Wolf, 2008; Grossmann and Grossmann, 2011; Petrowski et al., 2012). Furthermore, a few studies were able to show that the attachment style changes over the life span (Zhang and Labouvie-Vief, 2004; Scharfe and Cole, 2006; Wolf, 2008; Grossmann and Grossmann, 2011; Petrowski et al., 2012). Therefore, the attachment style may affect the style of living differently over the course of life and may not be the only influencing factor. Thus, the hypotheses posed by Schachner et al. (2008) could only partly be confirmed by the data for specific age periods.

Predicting the relationship status, individuals are *not single* if they do not mind depending on others, have high attachment anxiety, are older, and without higher education. However, high attachment anxiety was proposed for singlehood (Bookwala, 2003; Schachner et al., 2008; Adamczyk and Bookwala, 2013) and not as a predictor for being in a relationship. These results might be explicable since attachment anxiety or separation anxiety arises as soon as a partner is involved and, therefore, a new relationship might not be assumed. However, attachment style explained only 1% of the variance whereas the socio-demographic variables come to 40%. An income of below € 2000/month was linked to a nearly 13-fold increase of likelihood of remaining single. Thus, low income might indicate singlehood since a family cannot be supported on this income. In total, 41.1% of the variance of single adults could be explained by these variables.

## Merits, Limitations, and Prospects

The strength of this study is its large representative sample and the statistical approach to its results. However, a large sample size might easily lead to small but significant effects. To avoid this, we calculated effect sizes which were small to moderate. The present representative data are cross-sectional data. The single individuals were not in a relationship at this specific measurement point or age. However, whether these individuals had or will have a relationship cannot be concluded. But the strength of the study is that a wide age distribution was included. Furthermore, we used the original scale of the AAS (Collins and Read, 1994) and the instruction specific to romantic partners. The single individuals answered perhaps in a different or more “insecure” way because the scale is focused on a romantic partner, yet they may show great security in their friendships and other relationships which are not romantic relationships. With regard to the explained variance of approximately 41% it is clear that other variables have an influence on the relationship status. Therefore, earlier relationship experiences and the various attitudes toward partnership and “single-lifestyle” would be interesting factors in order to specify singlehood.

## Conclusion

In sum, the merit of the present study over previous studies is the large representative sample with a wide age distribution. The attachment style has a differential age-dependent association to singlehood versus a relationship. The older single individuals more often showed an anxious attachment style. Younger coupled individuals showed higher attachment anxiety. Education also played a role, exclusively concerning faith in others. More highly educated individuals showed fewer problems with dependency independent of singlehood or being in a relationship, and the single individuals were more highly educated. By predicting the relationship status, attachment style explained only 1% of the variance whereas the socio-demographic variables came to 40%. Most of the variance can be explained by socio-demographic variables.

## Author Contributions

KP, SS, and YSR drafted the work and revised it critically for important intellectual content. They also contributed substantially to the analysis and interpretation of the data. EB and GS contributed substantially to the conception and design of the work as well as the acquisition, analysis, and interpretation of data for the study. Both revised the work critically for important intellectual content. All Authors finally approved this version to be published.

### Conflict of Interest Statement

The authors declare that the research was conducted in the absence of any commercial or financial relationships that could be construed as a potential conflict of interest.
