# Erratum: Is It Attachment Style or Socio-Demography: Singlehood in a Representative Sample

**DOI:** 10.3389/fpsyg.2015.01946

**Published:** 2015-12-14

**Authors:** Frontiers Production Office

**Affiliations:** Frontiers Production Office, FrontiersSwitzerland

**Keywords:** adult attachment scale, attachment style, partnership status, representative sample, single person

Reason for Erratum:

Due to a misunderstanding the Acknowledgment Section was missing from the published article. The publisher apologizes for this error and the original article has been updated.

This error does not change the scientific conclusions of the article in any way.

